# Comparison of volume-controlled, pressure-controlled, and pressure-controlled volume-guaranteed ventilation during robot-assisted laparoscopic gynecologic surgery in the Trendelenburg position

**DOI:** 10.7150/ijms.49253

**Published:** 2020-09-23

**Authors:** Jung Min Lee, Soo Kyung Lee, Chae Chun Rhim, Kwon Hui Seo, Minsu Han, So Youn Kim, Eun Young Park

**Affiliations:** 1 Department of Anesthesiology and Pain Medicine, Hallym University Sacred Heart Hospital, College of Medicine, Hallym University, Anyang, Republic of Korea; 2 Department of Obstetrics and Gynecology, Hallym University Sacred Heart Hospital, College of Medicine, Hallym University, Anyang, Republic of Korea

**Keywords:** Mechanical ventilation, Trendelenburg position, general anesthesia, gynecologic surgery

## Abstract

**Background**: Pressure-controlled ventilation volume-guaranteed (PCV-VG) is being increasingly used for ventilation during general anesthesia. Carbon dioxide (CO_2_) pneumoperitoneum in the Trendelenburg position is routinely used during robot-assisted laparoscopic gynecologic surgery. Here, we hypothesized that PCV-VG would reduce peak inspiratory pressure (Ppeak), compared to volume-controlled ventilation (VCV) and pressure-controlled ventilation (PCV).

**Methods**: In total, 60 patients were enrolled in this study and randomly assigned to receive VCV, PCV, or PCV-VG. Hemodynamic variables, respiratory variables, and arterial blood gases were measured in the supine position 15 minutes after the induction of anesthesia (T0), 30 and 60 minutes after CO_2_ pneumoperitoneum and Trendelenburg positioning (T1 and T2, respectively), and 15 minutes after placement in the supine position at the end of anesthesia (T3).

**Results**: The Ppeak was higher in the VCV group than in the PCV and PCV-VG groups (p=0.011). Mean inspiratory pressure (Pmean) was higher in the PCV and PCV-VG groups than in the VCV group (p<0.001). Dynamic lung compliance (Cdyn) was lower in the VCV group than in the PCV and PCV-VG groups (p=0.001).

**Conclusion**: Compared to VCV, PCV and PCV-VG provided lower Ppeak, higher Pmean, and improved Cdyn, without significant differences in hemodynamic variables or arterial blood gas results during robot-assisted laparoscopic gynecologic surgery with Trendelenburg position.

## Introduction

Carbon dioxide (CO_2_) pneumoperitoneum and Trendelenburg positioning are commonly used to improve surgical access during robot-assisted laparoscopic gynecologic surgery. However, these methods are sometimes associated with several cardiopulmonary effects such as increased mean arterial pressure, decreased pulmonary compliance and functional residual capacity, increased peak inspiratory pressure (Ppeak), and respiratory acidosis in association with hypercarbia 1,2.

Volume-controlled ventilation (VCV) is the most commonly used method of ventilation during general anesthesia. It provides fixed minute ventilation and pulmonary resistance, which affect airway pressure 3. In pressure-controlled ventilation (PCV), constant inspiratory airway pressure can be achieved by decelerating the flow. However, minute ventilation is not fixed 4. Dual-controlled ventilation methods include pressure-controlled ventilation volume-guaranteed (PCV-VG), which has recently been introduced in the field of anesthesiology. Dual-controlled ventilation combines the advantages of VCV and PCV. It automatically calculates the pressure limits and delivers a preset tidal volume with the lowest required airway pressure 4-6.

CO_2_ pneumoperitoneum in the Trendelenburg position can influence hemodynamic variables, including blood pressure, heart rate, and cardiac output 2,7. This is because changes in airway pressure affect intrathoracic pressure and the function of the heart itself 8.

In this randomized study, we investigated the effects of VCV, PCV, and PCV-VG on Ppeak during robot-assisted laparoscopic gynecologic surgery involving CO_2_ pneumoperitoneum in the Trendelenburg position.

## Material and Methods

This study was approved by the Institutional Review Board of Hallym University Sacred Heart Hospital and registered at ClinicalTrials.gov (NCT 03887949). Written informed consent was obtained from all participants. Sixty patients scheduled for robot-assisted laparoscopic gynecologic surgery were enrolled. Patients were excluded if they had any of the following conditions: morbid obesity (body mass index > 30 kg/m²), hypotension (systolic blood pressure < 100 mmHg), bradycardia (heart rate < 60 bpm), history of heart failure, history of myocardial infarction, heart block, hypoxia (partial pressure of oxygen < 60 mmHg or peripheral oxygen saturation < 90%), uncontrolled asthma, or chronic obstructive pulmonary disease (forced expiratory volume in 1 s < 60%). Patients were also excluded if they were younger than 20 or older than 65 years of age.

The patients fasted for 8 hours before surgery and were premedicated with intramuscular glycopyrrolate (0.2 mg). Before the induction of anesthesia, patients were monitored by electrocardiography, non-invasive blood pressure, and pulse oximetry (peripheral oxygen saturation) in the operating room. The induction agents were intravenous remifentanil (0.1-0.2 μg/kg/min), propofol (1.5-2 mg/kg), and rocuronium (0.6 mg/kg). Anesthesia was maintained at a fractional inspired oxygen concentration of 0.5 with sevoflurane (2.0-2.5 vol%), remifentanil (0.05-0.3 μg/kg/min), and vecuronium (0.03-0.05 mg/kg/h). The patients were ventilated with an S/S AVANCE ventilator (Datex-Ohmeda; Madison, WI, USA) and randomly assigned to the VCV (n = 20), PCV (n = 20), or PCV-VG (n = 20) group by randomization software (http://www.randomlists.com). The tidal volume was set at 8 mL/kg of ideal body weight in all three groups. The respiratory rate (RR) was adjusted to maintain an end-tidal CO_2_ level of 30-38 mmHg, and the inspiratory to expiratory time ratio was 0.5. After induction of anesthesia, a 20-G catheter was inserted into the radial artery to monitor continuous arterial pressure, and connected to the FloTrac®/Vigileo system (Edwards Lifesciences Corp., Irvine, CA, USA) for continuous monitoring of cardiac output (CO), cardiac index (CI), stroke volume (SV), stroke volume index (SVI), and stroke volume variation (SVV).

Pneumoperitoneum with CO_2_ was induced with 12 mmHg of intraabdominal pressure and a 45° Trendelenburg position was established. Hemodynamic variables, respiratory variables, and arterial blood gases were measured in the supine position at 15 minutes after induction of anesthesia (T0), 30 and 60 minutes after CO_2_ pneumoperitoneum and Trendelenburg positioning (T1 and T2), and 15 minutes after placement in the supine position at the end of anesthesia (T3). The measured hemodynamic variables included mean arterial pressure, heart rate, CO, CI, SV, SVI, and SVV. Respiratory variables included the RR, Ppeak, mean inspiratory pressure (Pmean), peripheral oxygen saturation, end-tidal CO_2_ (ETCO_2_), and dynamic lung compliance (Cdyn).

The sample size required to detect a 15% difference in Ppeak between the VCV and PCV-VG groups (α = 0.05, power = 90%, effect size = 0.55) was determined in accordance with a previous study that compared three modes of ventilation during bariatric surgery 6. Assuming a dropout rate of 20%, 20 patients were included in each group.

Statistical analyses were performed using SPSS Statistics (version 26.0; IBM Corp., Armonk, NY, USA). The normality of the data distribution was assessed using the Shapiro-Wilk test. Normally distributed longitudinal data were analyzed using repeated-measures analysis of variance. Non-normally distributed variables data were analyzed using the generalized estimating equation approach. At each time point, differences among groups in normally distributed hemodynamic and respiratory data were analyzed using analysis of variance. Non-normally distributed data were analyzed with the Kruskal-Wallis test, and the Mann-Whitney U test with Bonferroni correction was applied for multiple comparisons. The paired t-test or Wilcoxon signed-rank test was used to analyze T0 variables in each group. The data are presented as mean ± standard deviation for normally distributed variables, and as median [interquartile range] for non-normally distributed variables. P-values <0.05 were considered statistically significant.

## Results

**Figure 1:**
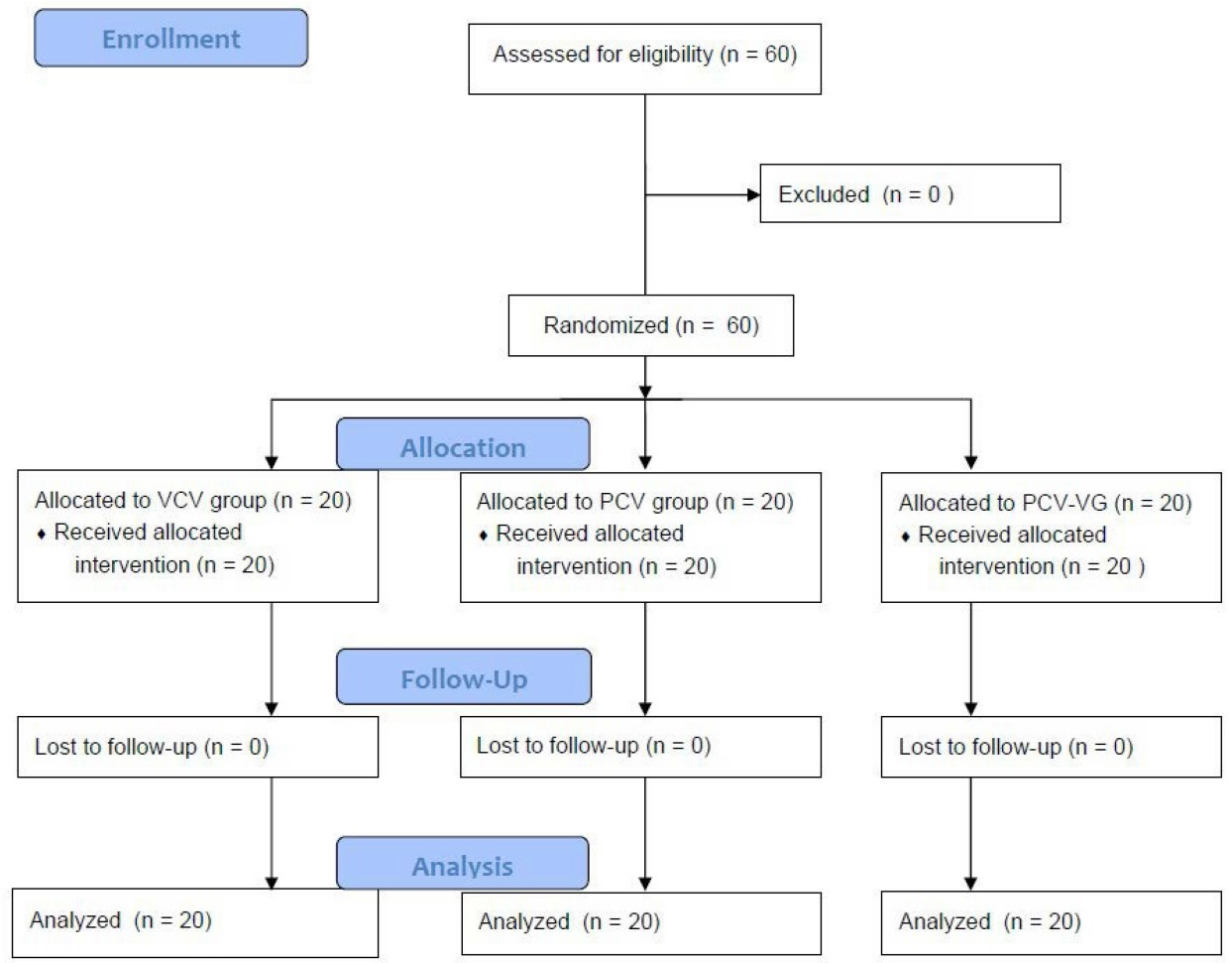
CONSORT flow diagram. VCV, volume-controlled ventilation; PCV, pressure-controlled ventilation; PCV-VG, pressure-controlled ventilation volume-guaranteed.

In total, 60 patients were enrolled in this study. All patients completed the study. The CONSORT flow diagram is shown in Figure 1. There were no significant differences among groups in terms of demographic data (Table 1).

**Figure 2:**
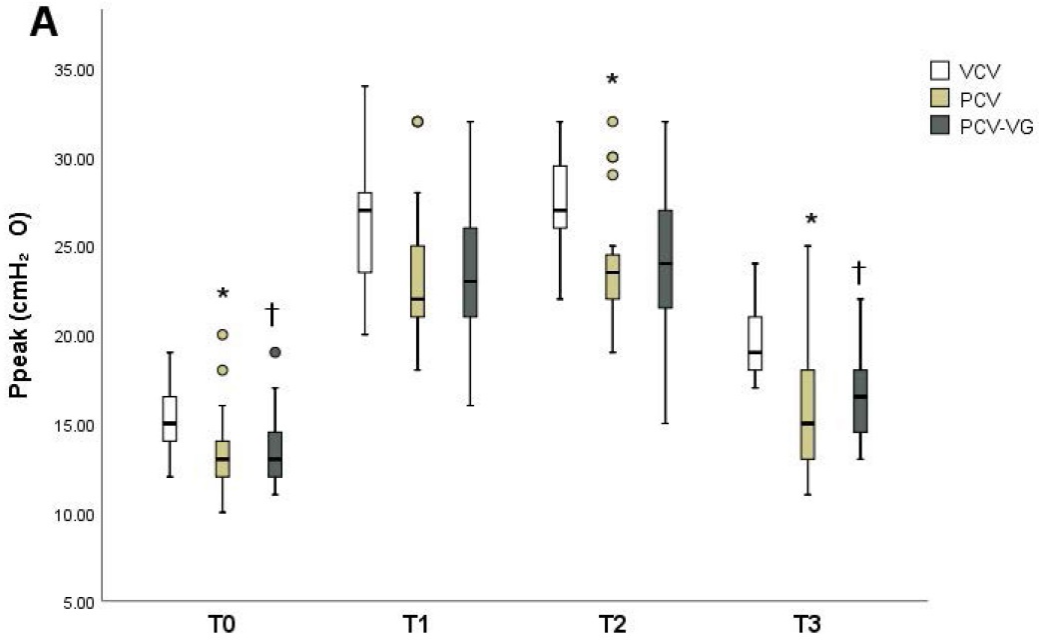

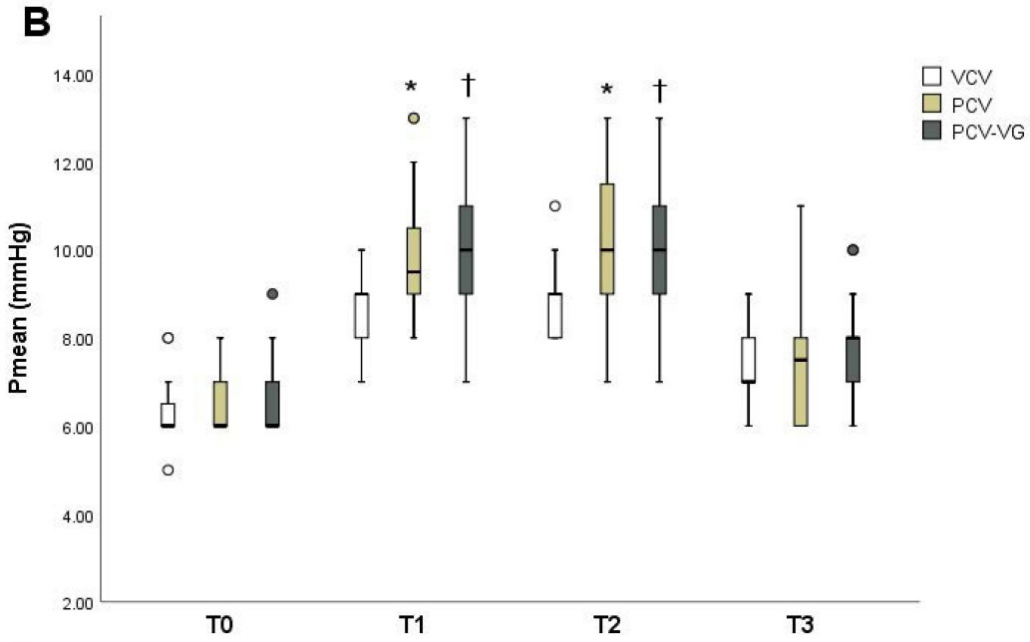

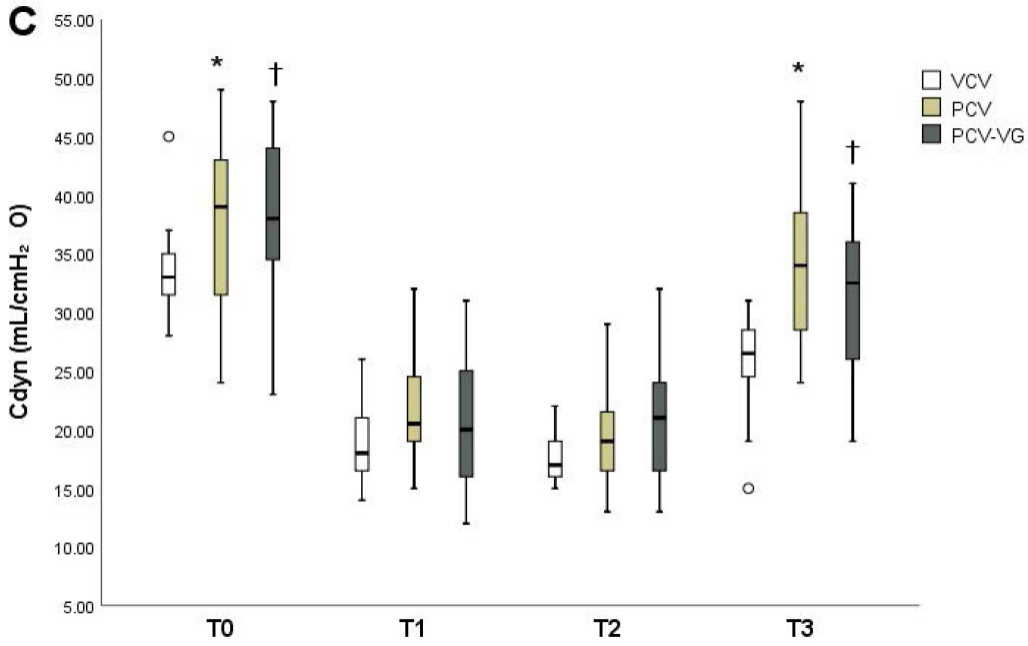
Changes in peak inspiratory pressure (Ppeak) (A), mean inspiratory pressure (Pmean) (B), and dynamic lung compliance (Cdyn) (C) at each measurement timepoint in each group. Data are shown as ranges, medians, and interquartile ranges. VCV, volume-controlled ventilation; PCV, pressure-controlled ventilation; PCV-VG, pressure-controlled ventilation volume-guaranteed; T0, in the supine position 15 minutes after the induction of anesthesia; T1, 30 minutes after CO₂ pneumoperitoneum and Trendelenburg positioning; T2, 60 minutes after CO₂ pneumoperitoneum and Trendelenburg positioning; T3, 15 minutes after placement in the supine position at the end of anesthesia. *P<0.016 for VCV vs. PCV at the same time point. †P<0.016 for VCV vs. PCV-VG at the same time point.

Ppeak, Pmean, and Cdyn showed significant differences among groups over time (Tables 2-4). The PCV group showed lower Ppeak (Figure 2A) at T0, T2, and T3 compared to the VCV group (p=0.006, p=0.001 and p=0.001, respectively). The PCV-VG group showed lower Ppeak values than the VCV group, which were significantly lower at T0 and T3 (p=0.015 and p<0.001, respectively). Pmean values (Figure 2) were higher in the PCV and PCV-VG groups than in the VCV group at T1 and T2 (T1: p=0.003 and p=0.001, respectively; T2: p=0.008 and p=0.003, respectively). Improved Cdyn values (Figure 2) were observed in the PCV and PCV-VG groups compared to the VCV group, which were significant higher at T0 (p=0.004 and p=0.002, respectively) and T3 (p=0.012 and p=0.001, respectively). Arterial blood gases and other hemodynamic and respiratory variables did not demonstrate significant differences among the three groups over time.

## Discussion

In this study, we compared the effects of VCV, PCV, and PCV-VG on pulmonary and hemodynamic variables in the Trendelenburg position with CO_2_ pneumoperitoneum. The PCV and PCV-VG groups showed lower Ppeak, higher Cdyn, and higher Pmean.

In VCV, the ventilator delivers a constant preset tidal volume, and airway pressure is influenced by pulmonary compliance 3,4. PCV provides a constant preset tidal volume by decelerating the flow, thereby achieving the desired tidal volume at lower Ppeak 9. However, the tidal volume can vary as a result of decreased lung compliance, especially during transition from the supine to the Trendelenburg position, or during induction of pneumoperitoneum with CO_2_. Furthermore, there is a risk of hypoventilation or hyperventilation 9,10. PCV-VG provides a target tidal volume by decelerating the flow, similar to PCV. It compares the Cdyn measured at each breath and adjusts inspiratory pressure to reach the set tidal volume 4,11.

Combining Trendelenburg positioning with CO_2_ pneumoperitoneum causes increased intrathoracic pressure 12,13, decreased lung compliance, increased Ppeak, and atelectasis 14. Several studies have compared VCV and pressure-mediated ventilation (PCV or dual-controlled ventilation) during laparoscopic surgeries with Trendelenburg positioning; their results are consistent with our findings of lower Ppeak and higher Cdyn values in pressure-mediated ventilation 10,11,15-18. Although the relationship between high Ppeak and respiratory consequence is controversial, Park et al. reported that their dual-controlled ventilation group showed a lower incidence of postoperative fever compared to the VCV group 11. A recent study concluded that there was no clear evidence of an association between atelectasis and fever; the authors proposed that different ventilation modes might affect the inflammatory response 11,19. Choi et al. reported that levels of biomarkers of acute lung injury were higher in their VCV group than in the PCV group. Moreover, there were significant correlations between Ppeak and the biomarkers 18, indicating that pressure-mediated ventilation with decelerating inspiratory flow is more beneficial than VCV in terms of protection against lung injury and reducing of inflammatory reactions.

The effects of Trendelenburg positioning with CO_2_ pneumoperitoneum on CI are variable. CI might be increased in this circumstance due to increased ventricular filling pressure. Increased intraabdominal pressure also improves venous return and splanchnic flow 2,16. Decreased CI is associated with impact of anesthesia 7,20, increased systemic vascular resistance in pneumoperitoneum 21 and the effects of intra-abdominal pressure on the heart 22. In the present study, CO and CI were lower at T2 than at T0 in all groups. These lower values were caused by decreased SV and SVI values at T2 (Table 3). Decreased preload during surgery due to inadequate fluid administration may have caused the decreased SV, because SVV was also higher at T2 than at T0. Because those parameters improved in the supine position at the end of anesthesia (T3), volume status and the increased systemic vascular resistance associated with CO_2_ pneumoperitoneum might have affected cardiac function.

We expected that Ppeak would affect pleural pressure by changing cardiac function 8. However, we found no significant differences in hemodynamic variables among the groups over time. Balick-Weber et al. 23 compared the effects cardiac function between VCV and PCV groups using transesophageal echocardiography. They found no echocardiographic differences between the groups and therefore concluded that Ppeak was not associated with cardiac function.

Pressure-mediated ventilation with decelerating flow delivers the bulk of the tidal volume during the initial phase of respiratory cycle, and increases the Pmean 24. Decelerating flow and higher Pmean are associated with improved oxygenation 24,25. Pmean values were higher in the PCV and PCV-VG groups than in the VCV group in our study. However, oxygenation did not improve significantly in the PCV or PCV-VG groups compared to the VCV group. PCV-VG improved oxygenation in studies of patients with obesity and patients who underwent one-lung ventilation 17,26. However, pressure-mediated ventilation did not lead to better oxygenation than VCV, despite the increased Pmean seen in the Trendelenburg position with CO_2_ pneumoperitoneum 11,15,16,18,23. Pmean values were significantly higher in the PCV and PCV-VG groups than in the VCV group in the present study, but clinically low Pmean seemed to have a minimal effect in terms of improving oxygenation.

The present study had several limitations. First, the investigators could not be blinded because they were aware of the ventilation modes. Second, patients with compromised cardiac or pulmonary diseases, as well as those with morbid obesity, were excluded. Because hemodynamics and respiratory mechanics might be affected by Trendelenburg positioning and CO_2_ pneumoperitoneum, we aimed to standardize cardiac and pulmonary function among the patients. The effects of pressure-mediated ventilation on oxygenation in patients with morbid obesity are unclear. Several studies have reported improved oxygenation with PCV and PCV-VG 17,27, but a meta-analysis concluded that there was no evidence of an effect of ventilation mode on oxygenation 28. Further studies are needed, including larger samples of patients strictly categorized according to cardiac and pulmonary function.

## Conclusion

PCV and PCV-VG were associated with lower Ppeak, higher Pmean, and improved Cdyn during robot-assisted laparoscopic gynecologic surgery in the Trendelenburg position compared to VCV. There was no significant group difference in CO, CI, SV, SVV, or oxygenation. There was no significant difference between the PCV and PCV-VG groups in Ppeak, Pmean, or Cdyn. However, PCV-VG may be an effective alternative to PCV during robot-assisted laparoscopic gynecologic surgery in the Trendelenburg position, during which lung compliance continuously varies, because a constant tidal volume can be achieved with the requirement for fewer adjustments compared to PCV.

## Figures and Tables

**Table 1:** Demographic characteristics

	VCV (n = 20)	PCV (n = 20)	PCV-VG (n = 20)
Age (years)	48.7±8.79	47.0±6.96	46.95±6.26
BMI (kg/m²)	24.17±3.04	24.48±2.87	23.89±2.34
Duration of anesthesia (min)	168.75±32.15	168.0±36.36	172.0±40.37
Duration of surgery (min)	121.50±33.37	120.0±33.86	119.75±34.36

Data are shown as mean ± standard deviation. BMI, body mass index; VCV, volume-controlled ventilation; PCV, pressure-controlled ventilation; PCV-VG, pressure-controlled ventilation volume-guaranteed.

**Table 2:** Respiratory variables

	Groups	T0	T1	T2	T3	ªP value
Ppeak (cmH₂O)	VCV	15 [14-16]	‡26 [24-28]	‡27 [26-29]	‡19 [18-20.5]	0.011
	PCV	*13 [12-13.5]	‡22 [21-24.5]	*‡23.5 [22-24]	*‡15 [13-17.5]	
	PCV-VG	†13 [12-14.5]	‡23 [21.5-25.5]	‡24 [22-27]	†‡16.5 [15-18]	
Pmean (cmH₂O)	VCV	6 [6-6]	‡9 [8-9]	‡9 [8-9]	‡7 [7-8]	<0.001
	PCV	6 [6-7]	*‡9.5 [9-10]	*‡10 [9-11]	‡7.5 [6-8]	
	PCV-VG	6 [6-7]	†‡10 [9-11]	†‡10 [9-11]	‡8 [7-8]	
RR (breaths/min)	VCV	12 [11-12]	‡12.5 [12-13]	‡13 [12-13]	‡13 [12-13]	0.065
	PCV	12 [12-12]	‡13.5 [13-14]	‡13 [12-14]	‡12.5 [12-14]	
	PCV-VG	12 [11-12]	‡12 [12-13]	‡12 [12-13]	‡12 [11-12]	
Cdyn (mL/cmH₂O)	VCV	33 [32-35]	‡18 [17-21]	‡17 [16-19]	‡27 [25-28]	0.001
	PCV	*39 [32-43]	‡21 [19-24]	‡19 [17-21]	*‡34 [29-38]	
	PCV-VG	†38 [35-44]	‡20 [16-25]	‡21 [17-24]	†‡33 [26-36]	
ETCO₂ (mmHg)	VCV	31 [30-32]	‡33 [32-34]	‡33 [32-34]	‡35 [33-36]	0.131
	PCV	31 [30-32.5]	‡34 [33-35]	‡34 [33-36]	‡34 [33-35]	
	PCV-VG	31 [30-33]	‡34 [33-35]	‡34 [33-35]	‡35 [34-36]	
SpO₂ (%)	VCV	99 [99-100]	99 [99-100]	99.5 [99-100]	100 [99-100]	0.790
	PCV	99 [99-100]	99 [98-100]	99 [99-100]	99.5 [99-100]	
	PCV-VG	99 [99-100]	99 [99-100]	99 [99-100]	99.5 [99-100]	

Data are shown as median [interquartile range]. Ppeak, peak inspiratory pressure; Pmean, mean inspiratory pressure; RR, respiratory rate; Cdyn, dynamic lung compliance; ETCO₂, end tidal CO₂; SpO₂, peripheral oxygen saturation; VCV, volume-controlled ventilation; PCV, pressure-controlled ventilation; PCV-VG, pressure-controlled ventilation volume-guaranteed; T0, in the supine position 15 minutes after the induction of anesthesia; T1, 30 minutes after CO₂ pneumoperitoneum and Trendelenburg positioning; T2, 60 minutes after CO₂ pneumoperitoneum and Trendelenburg positioning; T3, 15 minutes after placement in the supine position at the end of anesthesia. ªP values analyzed with generalized estimating equation. *P<0.016 for VCV vs. PCV at the same time point. †P<0.016 for VCV vs. PCV-VG at the same time point. ‡P<0.05 vs. T0 in each group.

**Table 3:** Hemodynamic variables

	Groups	T0	T1	T2	T3	ªP value
MAP (mmHg)	VCV	70±6.78	‡92.2±12.2	‡88.4±9.1	73.8±7.7	0.185
	PCV	73.7±13.7	‡93.4±13.0	‡86.3±8.6	70.2±8.2	
	PCV-VG	71.3±8.0	‡93.3±7.4	‡83.5±8.5	72.1±8.3	
HR (beats/min)	VCV	70.1±12.4	66.7±12.9	64.6±12.1	67.5±11.5	0.096
	PCV	67.3±10.3	68.9±9.5	64.0±6.9	65.4±7.3	
	PCV-VG	71.3±9.4	70.0±7.9	67.6±10.3	66.7±9.2	
CO (L/min/m²)	VCV	4.3 [3.7-4.7]	3.9 [3.3-4.8)	‡3.4 [3.1-4.0]	4.3 [3.8-4.9]	0.665
	PCV	4.5 [3.9-5.7]	4.1 [3.7-6.2)	‡3.9 [3.3-4.2]	4.6 [4.0-5.4]	
	PCV-VG	4.4 [4.0-5.5]	4.1 [3.7-4.7)	‡3.8 [3.6-4.3]	4.4 [4.1-4.7]	
CI (L/min/m²)	VCV	2.8 [2.2-3.2]	2.5 [2.1-3.1]	‡2.3 [1.9-2.8]	2.7 [2.4-3.1]	0.924
	PCV	2.8 [2.6-3.5]	2.7 [2.5-3.5]	‡2.4 [2.2-2.7]	2.9 [2.6-3.4]	
	PCV-VG	2.9 [2.6-3.4]	2.7 [2.4-3.1]	‡2.4 [2.2-2.8]	2.8 [2.7-3.0]	
SV (mL/beat)	VCV	62.5 [54.5-70]	60 [53-67]	55 [49-62]	65 [57-74]	0.858
	PCV	66 [60-77]	61 [54.5-75]	‡57 [53.5-71]	68 [62-78]	
	PCV-VG	64.5 [58-71]	58 [51-69]	‡55.5 [50-68]	66.5 [59-69]	
SVI (mL/m²/beat)	VCV	42 [35-44.5]	37 [34-47]	36.5 [32-40]	41 [37-48.5]	0.876
	PCV	42 [39-48.5]	41 [34.5-46.5]	‡38 [35-40]	44.5 [41-49]	
	PCV-VG	41 [37-46]	37 [33-45]	‡35.5 [33-41.5]	41.5 [38-46]	
SVV (%)	VCV	10.1±3.8	10.9±3.8	‡13.8±4.4	10.6±3.9	0.942
	PCV	9.4±3.5	9.8±4.3	‡13.3±5.5	9.9±4.2	
	PCV-VG	9.2±2.9	11.2±4.5	‡14.2±6.3	9.8±3.5	

Data are shown as mean ± standard deviation or median [interquartile range]. MAP, mean arterial pressure; HR, heart rate; CO, cardiac output; CI, cardiac index; SV, stroke volume; SVI, stroke volume index; SVV, stroke volume variation; VCV, volume-controlled ventilation; PCV, pressure-controlled ventilation; PCV-VG, pressure-controlled ventilation volume-guaranteed; T0, in the supine position 15 minutes after the induction of anesthesia; T1, 30 minutes after CO₂ pneumoperitoneum and Trendelenburg positioning; T2, 60 minutes after CO₂ pneumoperitoneum and Trendelenburg positioning; T3, 15 minutes after placement in the supine position at the end of anesthesia. ªP values analyzed with repeated-measures analysis of variance or generalized estimating equation. ‡P<0.05 vs. T0 in each group.

**Table 4:** Arterial blood gas analyses

	Groups	T0	T1	T2	T3	ªP value
PaO₂ (mmHg)	VCV	229.6±40.9	‡208.0±45.8	‡206.0±49.0	217.0±39.1	0.287
	PCV	207.0±49.1	‡189.2±46.7	198.4±43.6	200.6±43.5	
	PCV-VG	220.6±37.2	‡208.4±35.8	211.2±37.5	225.9±28.4	
PaCO₂ (mmHg)	VCV	34 [33-35]	‡36 [35-36]	‡36 [35-37]	‡37 [35-39]	0.093
	PCV	33 [33-35]	‡35 [34-36]	‡36 [35-38]	‡38 [36-39]	
	PCV-VG	34 [33-36]	‡36 [35-37]	‡36 [35-37]	‡36 [35-37]	
SaO₂ (%)	VCV	99.9 [99.8-100)	‡99.8 [99.7-99.9]	‡99.8 [99.7-99.9]	99.8 [99.7-99.9]	0.153
	PCV	99.8 [99.6-99.9)	99.7 [99.4-99.8]°	99.7 [99.6-99.8]	99.8 [99.5-99.9]	
	PCV-VG	99.9 [99.8-99.9)	‡99.8 [99.7-99.8]	‡99.8 [99.7-99.8]	99.8 [99.8-99.9]	

Data are shown as mean ± standard deviation or median [interquartile range]. PaO₂, partial pressure of oxygen; PaCO₂, partial pressure of carbon dioxide; SaO₂, arterial oxygen saturation; VCV, volume-controlled ventilation; PCV, pressure-controlled ventilation; PCV-VG, pressure-controlled ventilation volume-guaranteed; T0, in the supine position 15 minutes after the induction of anesthesia; T1, 30 minutes after CO₂ pneumoperitoneum and Trendelenburg positioning; T2, 60 minutes after CO₂ pneumoperitoneum and Trendelenburg positioning; T3, 15 minutes after placement in the supine position at the end of anesthesia. ªP values analyzed with repeated-measures analysis of variance or generalized estimating equation. ‡P<0.05 vs. T0 in each group.
